# IPH5201, an Anti-CD39 mAb, as Monotherapy or in Combination with Durvalumab in Advanced Solid Tumors

**DOI:** 10.1158/2767-9764.CRC-25-0361

**Published:** 2025-09-22

**Authors:** John Powderly, Martina Imbimbo, Antoine Italiano, Patricia Martin-Romano, Meredith McKean, Teresa Macarulla, Eduardo Castañón Alvarez, Benedito A. Carneiro, Raymond Mager, Vicky Barnhart, Steven Eck, Elina Murtomaki, Arsène-Bienvenu Loembé, Yun He, Zachary A. Cooper, Eric Tu, Chunling Fan, Agnes Boyer Chamard, Carine Paturel, Paula G. Fraenkel, Antoine Hollebecque

**Affiliations:** 1 Carolina BioOncology Institute, Huntersville, North Carolina.; 2 Oncology Department, Centre Hospitalier Universitaire Vaudois (CHUV), Lausanne University Hospital, Lausanne, Switzerland.; 3 Institut Bergonié, Bordeaux, France.; 4 Faculty of Medicine, University of Bordeaux, Bordeaux, France.; 5 Gustave Roussy Cancer Center, Villejuif, France.; 6 Sarah Cannon Research Institute, Nashville, Tennessee.; 7 Vall d’Hebron University Hospital, and Vall d’Hebron Institute of Oncology, Barcelona, Spain.; 8 Universidad de Navarra, Madrid, Spain.; 9 Legorreta Cancer Center, Brown University, and Lifespan Cancer Institute, Providence, Rhode Island.; 10 AstraZeneca, Gaithersburg, Maryland.; 11 AstraZeneca, Cambridge, United Kingdom.; 12 AstraZeneca, Waltham, Massachusetts.; 13 Innate Pharma, Marseilles, France.

## Abstract

**Purpose:**

Blocking enzymatic activity of cluster of differentiation 39 (CD39) with IPH5201 may promote antitumor immunity by increasing immunostimulatory ATP and reducing immunosuppressive adenosine levels in the tumor microenvironment. This first-in-human, phase 1 study evaluated IPH5201 as monotherapy or in combination with durvalumab (anti–PD-L1) in patients with advanced solid tumors.

**Patients and Methods:**

The study consisted of two consecutive dose-escalation parts: IPH5201 monotherapy (100, 300, 1,000, and 3,000 mg) every 3 weeks and IPH5201 (300, 1,000, and 3,000 mg) + durvalumab 1,500 mg every 3 weeks. The primary endpoint was to evaluate safety and tolerability. Secondary endpoints included antitumor activity, pharmacokinetics, and immunogenicity.

**Results:**

Overall, 38 patients received IPH5201 monotherapy and 19 received IPH5201 + durvalumab, with a median duration of follow-up of 7.6 months (range, 1.0–23.7). The most common cancer types were pancreatic (42.1%), non–small cell lung (19.3%), and colorectal (17.5%) cancers. The most common treatment-related adverse events were infusion-related reactions and fatigue. There were no adverse event–related deaths, and the maximum tolerated dose was not reached. Overall, 23/57 patients (40.4%) had stable disease as their best overall response. IPH5201 exhibited a linear pharmacokinetic profile and an estimated terminal half-life of 8 to 9 days at higher doses. On-treatment tumor biopsies revealed decreased CD39 ATPase activity in 5/7 patients, including all evaluable patients receiving the maximum dose of IPH5201 3,000 mg every 3 weeks.

**Conclusions:**

IPH5201 as monotherapy, or in combination with durvalumab, was well tolerated at pharmacologically active doses that induced reduction of intratumoral CD39 enzymatic activity and showed preliminary evidence for disease stabilization.

**Significance:**

The adenosine pathway is a source of emerging targets in cancer immunotherapy. IPH5201 is a mAb that targets the adenosine pathway by blocking human CD39 enzymatic activity. In this first-in-human, phase 1 study, IPH5201 as monotherapy or in combination with durvalumab was well tolerated with a manageable safety profile in patients with advanced solid tumors. Preliminary evidence for disease stabilization and reduction of tumoral CD39 enzymatic activity were observed.

## Introduction

Targeting the adenosine pathway (AP) is an emerging therapeutic strategy to promote antitumor immunity (1). Extracellular ATP may exhibit immune-stimulatory activity by binding the P2X and P2Y purinergic receptors, which promote activation of antigen-presenting cells in the tumor microenvironment (TME; ref. 2). Cluster of differentiation 39 (CD39) is an ectonucleotidase that hydrolyzes ATP to ADP and AMP. AMP is then degraded by CD73 to adenosine on the cell surface. This sequential activity of CD39 and CD73 generates immunosuppressive adenosine in the TME (3). Accumulation of adenosine elicits tumor-promoting effects, including dysregulating immune cell subsets, promoting vascularization, and impairing the function of immune cell infiltrates (4).

CD39 is known to be highly expressed in stromal, endothelial, and infiltrating immune cells of several tumor types, including non–small cell lung cancer (NSCLC), advanced pancreatic ductal adenocarcinoma (PDAC), renal cell carcinoma, and breast cancer (3, 5–11). Studies have shown that high expression of CD39 in the TME is associated with worse prognosis (10–12). Inhibition of CD39-mediated hydrolysis of ATP and ADP has the potential to promote antitumor immunity by inducing tumor accumulation of immunostimulatory ATP and reducing the formation of immunosuppressive adenosine (1, 4, 10).

IPH5201 is a humanized IgG1 mAb with a substantially reduced IgG effector function; IPH5201 selectively binds to and inhibits the activity of both the membrane-bound and soluble forms of CD39 (13). Five mutations—L234A, L235E, G237A, A330S, and P331S—are included in the Fc region of IPH5201 to substantially reduce IgG effector function (13). Preclinical data have shown that *in vitro*, IPH5201 enhanced phenotypic maturation and activation of dendritic cells and macrophages by inhibiting ATP hydrolysis. IPH5201 also restored T-cell proliferation to the levels observed in the absence of ATP addition (13). Durvalumab is a selective, high-affinity, human IgG1 mAb that binds to PD-L1 and blocks its interaction with PD-1 and CD80, thereby suppressing PD-L1–mediated inhibition of T-cell activation (14). Durvalumab has been shown to be clinically active as monotherapy or in combination with other agents across several solid tumor types (15–19).

We hypothesized that combination therapy with IPH5201 and durvalumab could increase the antitumor activity of durvalumab by altering the balance of ATP and adenosine in the TME. This is supported by preclinical murine tumor models in which CD39 blockade with IPH5201 or genetic deletion of CD39 increased the antitumor activity of an anti–PD-1 mAb or an ATP-inducing chemotherapeutic drug (3). In this study, we report the safety, pharmacokinetics (PK), immunogenicity, pharmacodynamics, and efficacy results of a first-in-human phase 1 study of IPH5201 as monotherapy or in combination with durvalumab in patients with advanced solid tumors.

## Materials and methods

### Patients

NCT04261075 was a first-in-human, multicenter, nonrandomized, open-label, phase 1 study. Full eligibility criteria are outlined in the Supplementary Appendix. In brief, eligible patients were ages ≥18 years, had histologically or cytologically confirmed advanced solid tumors, had at least one measurable lesion per RECIST v1.1, and had an Eastern Cooperative Oncology Group performance status of 0 or 1. Patients were refractory to standard therapy or had disease for which no standard therapy exists and consented to provide archival or fresh tumor samples. Patients may have received any conventional or investigational anticancer therapy (anti–cytotoxic T-lymphocyte–associated protein 4, anti–PD-1, and anti–PD-L1 antibodies), provided this was not within 21 days of the planned first dose of study treatment.

Key exclusion criteria included previous treatment with any agents targeting CD73, CD39, or adenosine receptors and active or prior autoimmune or inflammatory disorders within the past 5 years. Patients were also excluded if they had untreated metastases to the central nervous system or had cardiac and vascular comorbidities, including the presence of acute coronary syndrome or thromboembolic events within 6 months prior to enrollment, congestive heart failure, serious cardiac arrhythmia requiring medication, or uncontrolled hypertension.

All patients provided written informed consent prior to participation in the study. The study was carried out in accordance with the principles set out in the Declaration of Helsinki and consistent with the International Conference on Harmonization, Good Clinical Practice, and any applicable local laws and requirements. The protocol and all amendments were approved by the relevant institutional review boards or an independent Ethics Committee at each participating study center.

### Study design and treatment

The study consisted of two consecutive dose-escalation parts (Supplementary Fig. S1): ascending doses of intravenous IPH5201 monotherapy (100, 300, 1,000, and 3,000 mg), given every 3 weeks on day 1 of each cycle (part 1), or ascending doses of IPH5201 (300, 1,000, and 3,000 mg i.v.) in combination with intravenous durvalumab 1,500 mg every 3 weeks on day 1 of each cycle (part 2). Additional patients with PDAC or NSCLC were enrolled in part 1 or part 2 (IPH5201 dose levels 1,000 and 3,000 mg only) for pharmacodynamic analyses. A third part consisting of ascending dose levels of IPH5201 (300, 1,000, and 3,000 mg i.v.) in combination with intravenous durvalumab 1,500 mg every 3 weeks and intravenous oleclumab 3,000 mg every 3 weeks was planned but not initiated, because of a sponsor decision to terminate the trial that was unrelated to safety concerns.

In each part of the study, 3 to 12 patients received the planned doses in sequential cohorts. Once the maximum tolerated dose (MTD), recommended phase 2 dose, or highest protocol-defined dose (in the absence of MTD) in each part of the study had been determined, additional patients could be enrolled for further evaluation of the study endpoints. All patients received treatment until RECIST-defined disease progression or clinical progression, unacceptable toxicity, withdrawal of consent, or another protocol-defined discontinuation criterion was met. All patients were followed for survival until data cutoff or death (whichever occurred first).

### Endpoints and assessments

The primary endpoint was the safety and tolerability of IPH5201, assessed by the incidence of adverse events (AE), serious AEs (SAE) and dose-limiting toxicities (DLT), and to determine the MTD, recommended phase 2 dose, or highest protocol-defined dose (in the absence of MTD) of IPH5201, when administered as monotherapy or in combination with durvalumab. The DLT evaluation period during dose-escalation was 21 days from the first dose of study drug through the planned day of the second dose. A DLT was defined as any grade 3 or higher toxicity occurring during the DLT evaluation period. AEs that were clearly and directly related to the primary disease or to another etiology were excluded from this definition. DLTs are defined in full in the Supplementary Appendix.

Secondary endpoints included antitumor activity as measured by objective response rate (ORR) and disease control rate (DCR) per RECIST v1.1, determining the PK of IPH5201, and immunogenicity measured by the incidence of antidrug antibodies (ADA). ORR was defined as the proportion of patients with a best overall response (BOR) of confirmed complete response (CR) or confirmed partial response (PR). DCR was defined as the proportion of patients with a BOR of confirmed CR, confirmed PR, or having stable disease (SD) for a duration of ≥12 weeks.

Serum samples for determining IPH5201 PK were collected at planned time points following IPH5201 intravenous infusion. The concentration of IPH5201 in serum was determined by validated ELISA (see Supplementary Appendix).

Serum samples for detection of IPH5201 ADA were collected predose at planned time points and analyzed using a validated immunoassay following a tiered approach, in which clinical samples were tested in screening, confirmatory, and titer assays (see Supplementary Appendix).

Exploratory endpoints included progression-free survival (PFS), overall survival (OS), and assessment of biomarkers such as CD39 enzymatic activity, gene expression, and protein expression. The Wachstein–Meisel assay was used to detect the presence of phosphates hydrolyzed from ATP because of the enzymatic activity of CD39. CD39 enzymatic assay methods are described in full in the Supplementary Appendix. PFS was defined as the time from the first dose of treatment until the documentation of PD or death due to any cause, whichever occurred first. OS was defined as the time from the first dose of treatment until death due to any cause.

### Statistical methods

The safety evaluation was based on the as-treated population, defined as all patients who received any investigational product. AEs were coded using the Medical Dictionary for Regulatory Activities by system organ class and preferred term and graded according to the NCI Common Terminology Criteria for Adverse Events v5.0. Efficacy data were summarized based on the as-treated population. ORR and DCR were estimated with 95% confidence intervals (CI) using the exact probability method. The median PFS, OS, and their 95% CIs were estimated using the Kaplan–Meier method. PK evaluation was conducted in patients who received at least one dose of IPH5201 and provided at least one quantifiable posttreatment sample. Noncompartmental analysis for IPH5201 PK data was performed, and descriptive statistics of noncompartmental PK parameters were provided. For immunogenicity assessment, only patients who received at least one dose of IPH5201 and provided baseline and at least one posttreatment sample were evaluable for ADA assessment. Immunogenicity results were summarized descriptively by the number and percentage of patients who develop detectable anti-IPH5201 antibodies.

## Results

### Patients

A total of 75 patients were enrolled at eight study centers in four countries (United States, France, Spain, and Switzerland) from March 3, 2020, to July 29, 2021. Database lock was October 21, 2022. Of the 75 enrolled participants, 18 were screen failures due to not meeting the inclusion/exclusion criteria and did not receive treatment. The 57 remaining patients received study treatment, representing the as-treated population, of whom 38 received IPH5201 monotherapy and 19 received IPH5201 in combination with durvalumab, with a median duration of follow-up for all patients of 7.6 months (range, 1.0–23.7). Baseline demographics are presented in Table 1. The median age was 62 years (range, 41–76) in the IPH5201 monotherapy group and 64 years (range, 41–78) in the IPH5201 + durvalumab group. Most patients were male (IPH5201 monotherapy group, 52.6%; IPH5201 + durvalumab group, 57.9%) and both groups had a median of three prior therapies (range, IPH5201 monotherapy group, 2–10; IPH5201 + durvalumab group, 2–11). The most commonly enrolled patients in both groups had either PDAC (24 patients, 42.1%) or NSCLC (11 patients, 19.3%). All as-treated patients were PK-evaluable; 54 patients were ADA-evaluable. All patients had discontinued treatment at the end of the study, and 93% of patients discontinued IPH5201 because of disease progression.

**Table 1.** Patient characteristics^a^.

​	IPH5201					IPH5201 + durvalumab 1,500 mg			Total (*N* = 19)	Total (*N* = 57)
	100 mg (*n* = 3)	300 mg (*n* = 3)	1,000 mg (*n* = 13)	3,000 mg (*n* = 19)	Total (*N* = 38)	300 mg (*n* = 4)	1,000 mg (*n* = 8)	3,000 mg (*n* = 7)		
Median age (range), years	59.0 (48–60)	63.0 (50–71)	63.0 (46–76)	62.0 (41–75)	62.0 (41–76)	58.5 (48–73)	61.5 (47–71)	64.0 (41–78)	64.0 (41–78)	62.0 (41–78)
Male, *n* (%)	3 (100)	1 (33.3)	5 (38.5)	11 (57.9)	20 (52.6)	2 (50.0)	4 (50.0)	5 (71.4)	11 (57.9)	31 (54.4)
Race, *n* (%)^b^	​	​	​	​	​	​	​	​	​	​
White	3 (100)	3 (100)	13 (100)	17 (94.4)	36 (97.3)	4 (100)	7 (100)	7 (100)	18 (100)	54 (98.2)
Other	0	0	0	1 (5.6)	1 (2.7)	0	0	0	0	1 (1.8)
ECOG PS 0/1, %	66.7/33.3	33.3/66.7	7.7/92.3	47.4/52.6	34.2/65.8	100/0	37.5/62.5	14.3/85.7	42.1/57.9	36.8/63.2
Cancer diagnosis, *n* (%)	​	​	​	​	​	​	​	​	​	​
Pancreatic	1 (33.3)	0	9 (69.2)	8 (42.1)	18 (47.4)	1 (25.0)	5 (62.5)	0	6 (31.6)	24 (42.1)
Colorectal	0	2 (66.7)	1 (7.7)	2 (10.5)	5 (13.2)	0	1 (12.5)	4 (57.1)	5 (26.3)	10 (17.5)
NSCLC (squamous)	0	0	2 (15.4)	5 (26.3)	7 (18.4)	0	1 (12.5)	1 (14.3)	2 (10.5)	9 (15.8)
NSCLC (adenocarcinoma)	0	0	1 (7.7)	1 (5.3)	2 (5.3)	0	0	0	0	2 (3.5)
Other^c^	2 (66.7)	1 (33.3)	0	3 (15.8)	6 (15.8)	3 (75.0)	1 (12.5)	2 (28.6)	6 (31.6)	12 (21.1)
Median no. of prior lines of systemic therapy (range)	3.0 (2–3)	4.0 (3–8)	2.0 (2–10)	3.0 (2–8)	3.0 (2–10)	3.0 (2–5)	3.0 (2–4)	5.0 (2–11)	3.0 (2–11)	3.0 (2–11)

Abbreviations: ECOG PS, Eastern Cooperative Oncology Group performance status; NSCLC, non-small cell lung cancer.

a Data are shown based on the as-treated population, defined as all patients who received any investigational product.

b Each race category counted patients who selected only that category; two patients did not select a category and are not included in the table.

c Includes cervical [*n* = 1 (1.8%)], endometrial [*n* = 1 (1.8%)], ovarian [*n* = 2 (3.5%)], prostate [*n* = 2 (3.5%)], squamous cell carcinoma of the skin [*n* = 1 (1.8%)], thyroid cancer [*n* = 1 (1.8%)], and other cancers [*n* = 4 (7.0%)].

### Safety

The safety profile was reported in the as-treated population. The median duration of exposure to IPH5201 was 10.6 weeks (range, 3.0–48.0) in the IPH5201 monotherapy group and 8.7 weeks (range, 3.0–66.1) in the IPH5201 + durvalumab group. The median duration of exposure to durvalumab was 8.7 weeks (range, 3.0–66.1). Two patients discontinued treatment after one cycle (3 weeks) because of AEs, and three patients stopped treatment after one cycle because of disease progression.

Treatment-emergent AEs (TEAE) occurred in 36 of 38 patients (94.7%) in the IPH5201 monotherapy group and 19 of 19 patients (100%) in the IPH5201 + durvalumab group. Grade ≥3 TEAEs were reported in 17 of 38 patients (44.7%) and 6 of 19 patients (31.6%) in the monotherapy and combination therapy groups, respectively (Table 2). The most common any-grade TEAEs with frequency ≥20% in the IPH5201 monotherapy group were fatigue (26.3%), anemia (23.7%), infusion-related reactions, tumor pain, and dyspnea (21.1% each). In the IPH5201 + durvalumab group, the most common any-grade TEAEs were decreased appetite (42.1%), fatigue (31.6%), headache (26.3%), infusion-related reactions, nausea, and pyrexia (21.1% each; Supplementary Table S1). The most common grade ≥3 TEAEs with frequency ≥5% in the IPH5201 monotherapy cohort were anemia (13.2%), pulmonary embolism (10.5%), and embolism (5.3%). In the IPH5201 + durvalumab group, the following grade ≥3 TEAEs occurred in one patient (5.3%) each: anemia, colitis, fatigue, soft tissue infection, blood pressure increase, lipase increase, lymphocyte count decrease, back pain, muscular weakness, cerebrovascular accident, urinary tract obstruction, and embolism.

**Table 2.** Safety summary^a^.

​	IPH5201					IPH5201 + durvalumab 1,500 mg				Total (*N* = 57)
	100 mg (*n* = 3)	300 mg (*n* = 3)	1,000 mg (*n* = 13)	3,000 mg (*n* = 19)	Total (*N* = 38)	300 mg (*n* = 4)	1,000 mg (*n* = 8)	3,000 mg (*n* = 7)	Total (*N* = 19)	
Any TEAEs, *n* (%)	3 (100)	3 (100)	12 (92.3)	18 (94.7)	36 (94.7)	4 (100)	8 (100)	7 (100)	19 (100)	55 (96.5)
Grade ≥3 TEAEs^b^	1 (33.3)	0	7 (53.8)	9 (47.4)	17 (44.7)	1 (25.0)	2 (25.0)	3 (42.9)	6 (31.6)	23 (40.4)
Any TRAEs, *n* (%)	1 (33.3)	3 (100)	8 (61.5)	12 (63.2)	24 (63.2)	3 (75.0)	5 (62.5)	6 (85.7)	14 (73.7)	38 (66.7)
Related to IPH5201	1 (33.3)	3 (100)	8 (61.5)	12 (63.2)	24 (63.2)	2 (50.0)	5 (62.5)	6 (85.7)	13 (68.4)	37 (64.9)
Related to durvalumab	NA	NA	NA	NA	NA	3 (75.0)	4 (50.0)	4 (57.1)	11 (57.9)	11 (57.9)
Grade 3 TRAEs, *n* (%)^b^^,^^c^	0	0	1 (7.7)	3 (15.8)	4 (10.5)	1 (25.0)	0	1 (14.3)	2 (10.5)	6 (10.5)
Related to IPH5201	0	0	1 (7.7)	3 (15.8)	4 (10.5)	0	0	1 (14.3)	1 (5.3)	5 (8.8)
Related to durvalumab	NA	NA	NA	NA	NA	1 (25.0)	0	1 (14.3)	2 (10.5)	2 (10.5)
Treatment discontinuations	​									​
Discontinued IPH5201, *n* (%)	3 (100)	3 (100)	13 (100)	19 (100)	38 (100)	4 (100)	8 (100)	7 (100)^d^	19 (100)	57 (100)
Due to AEs	0	0	1 (7.7)	1 (5.3)	2 (5.3)	0	0	1 (14.3)	1 (5.3)	3 (5.3)
Due to progressive disease	3 (100)	3 (100)	12 (92.3)	18 (94.7)	36 (94.7)	4 (100)	8 (100)	5 (71.4)	17 (89.5)	53 (93.0)
Discontinued durvalumab, *n* (%)	NA	NA	NA	NA	NA	4 (100)	8 (100)	7 (100)^d^	19 (100)	19 (100)
Due to AEs	NA	NA	NA	NA	NA	0	0	1 (14.3)	1 (5.3)	1 (5.3)
Due to progressive disease	NA	NA	NA	NA	NA	4 (100)	8 (100)	5 (71.4)	17 (89.5)	17 (89.5)

Abbreviations: AE, adverse event; CTCAE, Common Terminology Criteria for Adverse Events; NA, not applicable; TEAE, treatment-emergent adverse event; TRAE, treatment-related adverse event.

a Data are shown based on the as-treated population, defined as all patients who received any investigational product.

b AEs were graded according to NCI CTCAE v5.0.

c There were no grade ≥4 TRAEs.

d One patient discontinued because of withdrawal of consent.

Treatment-related AEs (TRAE) occurred in 24 of 38 patients (63.2%) in the IPH5201 monotherapy group and 14 of 19 patients (73.7%) in the IPH5201 + durvalumab group. Any-grade TRAEs related to IPH5201 were reported in 24 of 38 patients (63.2%) and 13 of 19 patients (68.4%) in the monotherapy and combination therapy groups, respectively; 11 of 19 patients (57.9%) had TRAEs related to durvalumab (Table 2). Across both groups, 10.5% of patients had grade 3 TRAEs, and no grade 4 TRAEs were reported (Table 2). The most common any-grade TRAEs with a frequency ≥10% in the IPH5201 monotherapy group were infusion-related reactions (21.1%), fatigue (15.8%), pruritus (13.2%), nausea, and arthralgia (each 10.5%). In the IPH5201 + durvalumab group, the most common any-grade TRAEs with a frequency ≥10% were infusion-related reactions, fatigue (each 21.1%), pyrexia, decreased appetite (each 15.8%), tumor pain, asthenia, and flushing (each 10.5%; Supplementary Table S2). Grade ≥3 TRAEs observed in the IPH5201 monotherapy group were lymphopenia, atrioventricular block, biliary obstruction, and pulmonary embolism, occurring in one patient (2.6%) each. In the IPH5201 + durvalumab group, grade ≥3 TRAEs were colitis, fatigue, back pain, and embolism, occurring in one patient (5.3%) each.

One patient with endometrial cancer in the IPH5201 3,000 mg + durvalumab cohort experienced a DLT of grade 3 embolism (Medical Dictionary for Regulatory Activities Preferred Term) in the lung 20 days after the first dose of IPH5201 and durvalumab; the patient also had deep vein thrombosis in the femoral veins. The DLT was a grade 3 SAE, which led to discontinuation of both study treatments. This SAE was considered by the investigator to be related to IPH5201 and unrelated to durvalumab. No other SAEs led to discontinuation of study treatment (Table 2), and no deaths due to TRAEs occurred in the study. Any-grade thromboembolic AEs, including pulmonary embolism and vascular embolism, occurred in 11 of 57 patients (19.3%; Supplementary Table S3). The MTD was not reached.

### Efficacy

**Figure 1.**
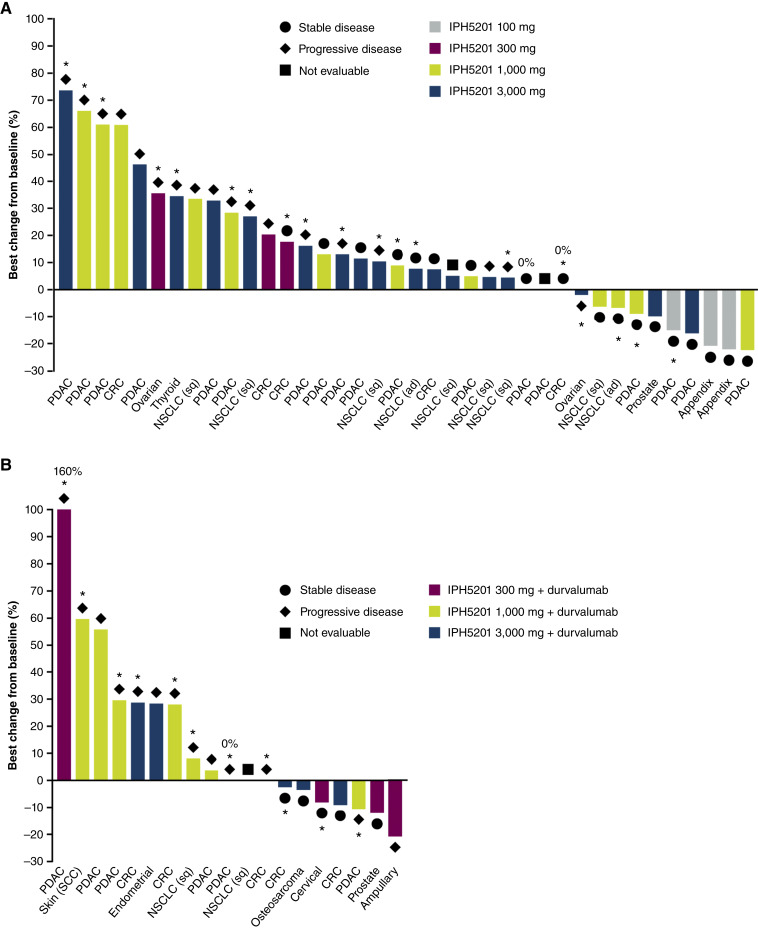
Best change in tumor size from baseline for (**A**) IPH5201 monotherapy (*n* = 38) and (**B**) IPH5201 + durvalumab (*n* = 19). Patients who developed new lesion as defined per RECIST v1.1 are denoted by an asterisk (*). Data are shown based on the as-treated population, defined as all patients who received any investigational product. ad, adenocarcinoma; CRC, colorectal cancer; NSCLC, non-small cell lung cancer; PDAC, pancreatic ductal adenocarcinoma; SCC, squamous cell carcinoma; sq, squamous.

In the as-treated population, 18 of 38 patients (47.4%) in the IPH5201 monotherapy group reported SD as their BOR over the course of the study [including 15 of 35 patients (42.9%) receiving ≥300 mg IPH5201], and 5 of 19 patients (26.3%) in the IPH5201 + durvalumab group reported a BOR of SD (Table 3). None had a BOR of CR or PR (Fig. 1). In the subgroup of patients with PDAC, a BOR of SD was reported in 9 of 24 patients (37.5%), and PD was reported in 14 of 24 patients (58.3%; monotherapy and combination groups combined). In patients with NSCLC, a BOR of SD was reported in 3 of 11 patients (27.3%), and PD was reported in 6 of 11 patients (54.5%) patients (monotherapy and combination groups combined). A reduction in the total sum of diameters of target lesions was seen in 26.3% (10/38) of patients treated with IPH5201 monotherapy and 36.8% (7/19) of patients treated with combination therapy across all doses (Fig. 1). The DCR at 12 weeks was 36.8% in the IPH5201 monotherapy group and 26.3% in the IPH5201 + durvalumab group (Table 3). The median PFS was 2.4 months (95% CI, 1.4–3.5) with IPH5201 monotherapy and 1.4 months (95% CI, 1.3–1.4) with IPH5201 + durvalumab, with 91.2% data maturity across the groups (Table 3); the median OS was 7.8 (95% CI, 5.9–10.9) and 9.2 (95% CI, 3.0–not calculated) months in the monotherapy and combination groups, respectively, with 70.2% data maturity across the groups (Table 3).

**Table 3.** Summary of efficacy^a^.

​	IPH5201					IPH5201 + durvalumab 1,500 mg				Total (*N* = 57)
	100 mg (*n* = 3)	300 mg (*n* = 3)	1,000 mg (*n* = 13)	3,000 mg (*n* = 19)	Total (*N* = 38)	300 mg (*n* = 4)	1,000 mg (*n* = 8)	3,000 mg (*n* = 7)	Total (*N* = 19)	
BOR, *n* (%)	​	​	​	​	​	​	​	​	​	​
SD	3 (100)	1 (33.3)	8 (61.5)	6 (31.6)	18 (47.4)	2 (50.0)	0	3 (42.9)	5 (26.3)	23 (40.4)
PD	0	2 (66.7)	5 (38.5)	11 (57.9)	18 (47.4)	2 (50.0)	8 (100)	3 (42.9)	13 (68.4)	31 (54.4)
NE	0	0	0	2 (10.5)	2 (5.3)	0	0	1 (14.3)	1 (5.3)	3 (5.3)
Disease control ≥12 weeks, *n* (%)	3 (100)	1 (33.3)	6 (46.2)	4 (21.1)	14 (36.8)	2 (50.0)	0	3 (42.9)	5 (26.3)	19 (33.3)
Median PFS, months (95% CI)	NC (4.1–NC)	1.3 (1.3–NC)	2.5 (1.3–5.3)	1.4 (1.3–2.8)	2.4 (1.4–3.5)	6.9 (1.4–NC)	1.3 (0.8–1.4)	1.4 (0.7–6.2)	1.4 (1.3–1.4)	1.4 (1.4–2.5)
Median OS, months (95% CI)	12.5 (9.8–NC)	13.1 (1.6–NC)	8.2 (4.4–8.7)	7.0 (4.3–11.2)	7.8 (5.9–10.9)	NC (2.1–NC)	4.2 (2.7–NC)	11.2 (1.0–NC)	9.2 (3.0–NC)	8.2 (5.9–10.3)

Abbreviations: BOR, best overall response; CI, confidence interval; NC, not calculated; NE, not evaluable; PD, progressive disease; SD, stable disease.

a Data are shown based on the as-treated population, defined as all patients who received any investigational product.

### PK

**Figure 2.**
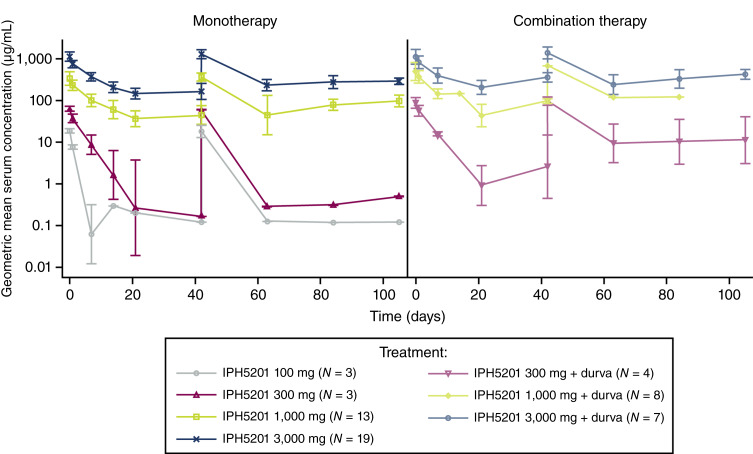
Mean (± SD) serum concentration of IPH5201 across dose cohorts and treatment. Data are shown based on the PK analysis set, defined as patients who received ≥1 dose of IPH5201 or durvalumab (durva) and provided ≥1 quantifiable posttreatment sample. Vertical lines represent the geometric ± SD. Geometric mean SD was calculated as the exponential [mean (log (PK concentration)) ± SD (log (PK concentration))]. PK, pharmacokinetic; SD, standard deviation.

Following a single dose, IPH5201 exhibited a nonlinear PK profile at 100 and 300 mg and a linear PK profile at doses of 1,000 mg and higher. The increase in exposure to IPH5201 was more than proportional to the increase in dose for both monotherapy treatment and when given in combination with durvalumab, except for the increase from 1,000 to 3,000 mg which seemed generally proportional (Fig. 2; Supplementary Table S4), indicating target-mediated drug distribution. The estimated half-life associated with terminal slope (t_1/2ʎz_) seemed to increase with dose and was approximately 8 to 9 days at IPH5201 doses ≥1,000 mg. Following repeat administration, there was no apparent evidence of accumulation based on C_max_, in which geometric mean values of accumulation ratio were generally around 1 (Supplementary Table S4).

### Immunogenicity

The prevalence of IPH5201 ADAs (i.e., the percentage of ADA-evaluable patients with a positive ADA result at any time) was 37/54 (68.5%). There were no treatment-boosted ADAs, and 33 patients were positive for treatment-induced ADA; therefore ADA incidence (i.e., the percentage of ADA-evaluable patients who were treatment-emergent ADA-positive) was 33/54 (61.1%). Nine patients (16.7%) were classified as ADA persistently positive (i.e., positive for treatment-emergent ADA and had at least two postbaseline ADA-positive measurements with >16 weeks between the first and last positive measurements; Supplementary Table S5). There was no apparent trend in IPH5201 exposure related to ADA.

### Pharmacodynamics

#### Receptor occupancy

IPH5201 saturated binding of soluble CD39 in sera at ≥300 mg (Supplementary Fig. S2) and binding of membrane-bound CD39 on monocytes and B cells at 3,000 mg (Supplementary Fig. S2). Free soluble CD39 was detected in sera above the lower limit of quantitation at cycle 1, day 2 or later in 6/40 patients (*n* = 2, 100 mg; *n* = 1, 1,000 mg; *n* = 3, 3,000 mg). Similar trends were observed when IPH5201 was combined with durvalumab.

#### CD39 enzymatic activity

**Figure 3.**
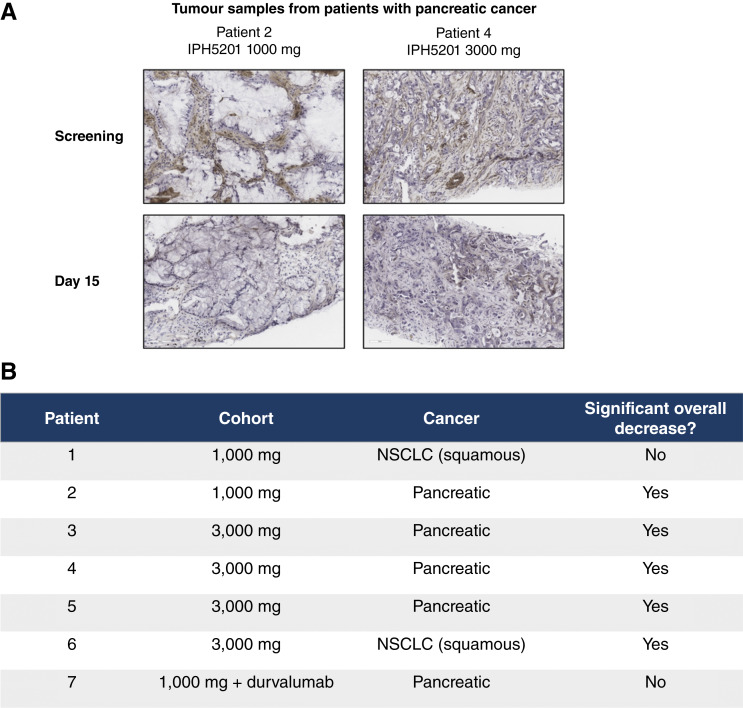
IPH5201 treatment decreases tumoral CD39 enzymatic activity. The Wachstein–Meisel assay detects the presence of phosphates hydrolyzed from ATP due to enzymatic activity of CD39. **A,** Representative histology images of tumor tissues with reduction in CD39 enzymatic activity after treatment with IPH5201. **B,** Enzymatic activity was assessed by semi-quantitative scoring methodology for tumor, stroma, and vasculature. The pathologist conducted an overall assessment to determine whether a significant decrease was present on day 15 compared with screening biopsies.

Semi-quantitative scoring of CD39 enzymatic activity in patient tumor samples was used to assess correlation with antitumor activity. IPH5201 treatment reduced tumoral CD39 enzymatic activity in five out of seven patients who showed baseline enzymatic CD39 activity and had samples available before and after treatment (Fig. 3). All five patients with reduced enzymatic activity received IPH5201 monotherapy; four patients had PDAC, and one had squamous NSCLC. Four of these five patients received the maximum IPH5201 dose of 3,000 mg; the remaining patient received IPH5201 1,000 mg. Both the patients who did not show a significant overall decrease in enzymatic activity received IPH5201 1,000 mg, suggesting that a IPH5201 dose of 3,000 mg is required to ensure complete blockage of tumoral CD39 enzymatic activity (Fig. 3).

## Discussion

This report provides the first-in-human clinical safety and efficacy data for IPH5201, an anti-CD39 mAb, administered as monotherapy or in combination with the anti–PD-L1 mAb durvalumab. IPH5201 treatment with or without durvalumab was well tolerated at pharmacologically active doses that induced substantial reduction of intratumoral CD39 enzymatic activity and showed preliminary evidence for disease stabilization in patients with advanced solid tumors.

The safety profile of IPH5201 alone and in combination with durvalumab was manageable. There were no new safety signals identified beyond those observed with durvalumab monotherapy. Similar safety profiles have been reported for other therapies in clinical development that target the AP, including anti-CD39 mAbs. In a first-in-human, phase 1 study of SRF617, a human IgG4 anti-CD39 antibody (20), SRF617 was administered as monotherapy or in combinations with gemcitabine plus albumin-bound paclitaxel (nab-paclitaxel) or the PD-L1 inhibitor, pembrolizumab, in 55 patients with advanced solid tumors (21). SRF617 was well tolerated at all dose levels with a manageable safety profile and no DLTs (21).

CD39 is known to play a key role in limiting thrombotic events and regulating platelet function (22, 23). Although CD39-deficient mice have altered platelet function (22), preclinical studies have shown that anti-CD39 administration in mice did not promote thrombosis (24, 25). Of note, the current overall study population was enriched for patients with PDAC (24 of 57 patients, 42.1%), and any-grade thromboembolic AEs were observed more frequently in this cohort of patients (8 of 24 patients, 33.3%). PDAC is known to carry the highest risk of thromboembolic events among all cancers (26); a retrospective study in advanced pancreatic cancer identified venous thromboembolic events in 25% of patients (27). Other published studies have reported rates between 10% and 35% in patients with pancreatic cancer (28–31). Thus, the overall incidence of thromboembolic events observed in the present study (19.3%; Supplementary Table S3) seems similar to previously published rates in this population, although direct comparisons are difficult due to differences in sample size, study design, and cohort selection.

These phase 1 data for IPH5201 add to the emerging knowledge gained from studies of other related therapeutics in clinical development, including SRF617, oleclumab (an anti-CD73 antibody), and other agents targeting the adenosine–A2A receptor pathway (10). Both SRF617 and IPH5201 have shown a tendency toward disease stabilization in their respective phase 1 trials. In the SRF617 phase 1 study, one confirmed PR (14.2%, 1/7) was observed in a patient with PDAC in combination with gemcitabine and nab-paclitaxel (21). In the SRF617 monotherapy group, 31.3% (10/32) patients exhibited SD at 8 weeks; in the SRF617 and pembrolizumab combination therapy group, 50% (4/8) patients demonstrated SD at 6 weeks (21). In the phase 2, multidrug NeoCOAST study of patients with resectable NSCLC, pathologic CR occurred in 9.5% (2/21) of patients who were treated with neoadjuvant durvalumab in combination with oleclumab (32), supporting the hypothesis that targeting the AP enhances the efficacy of immune checkpoint blockade.

In the current study, IPH5201 exhibited a PK profile of target-mediated drug disposition with the increase in exposure generally proportional from 1,000 to 3,000 mg, consistent with the receptor occupancy of IPH5201 which saturated binding of soluble CD39 in sera and binding of membrane-bound CD39 on monocytes and B cells. Although induction of ADA was frequent, it did not seem to impair exposure. IPH5201 was pharmacologically active, with the 3,000 mg dose resulting in blockade of tumoral CD39 enzymatic activity. The evidence for IPH5201’s pharmacodynamic activity rests on the inhibition of CD39 activity in on-treatment tumor specimens compared with baseline. The current analysis is more comprehensive than that previously reported for SRF617, which demonstrated decreased CD39 expression on IHC in a single patient’s on-treatment biopsy compared with baseline (21). The Wachstein–Meisel assay (33) is a more precise way to assess the pharmacodynamic activity than by assessment of CD39 expression level, as IPH5201 does not cause CD39 internalization or down modulation (3). Analysis with this assay demonstrated decreased CD39 ATPase activity in 5/7 patients, including all evaluable patients receiving the maximum dose of IPH5201 3,000 mg every 3 weeks.

Limitations of this phase 1 study include the small number of patients enrolled, with 38 and 19 patients in the monotherapy and combination treatment groups, respectively. The study enrollment focused on four indications of interest, namely PDAC, colorectal cancer, squamous NSCLC, and adenocarcinoma NSCLC, with a bias toward PDAC (47.4% and 31.6% of patients in the monotherapy and combination therapy groups, respectively). Although IPH5201 treatment led to SD in many patients, there was no CR or PR. The ADA assay may require further refinement, as the majority of the 37 patients who were ADA-positive had low titers. Nevertheless, the clinical and safety data presented here enrich our understanding of the potential role for targeting CD39 in antineoplastic therapies.

To realize the full potential of AP-targeting agents, combination with antineoplastic agents that induce immunogenic cell death may be needed. Preclinical experiments in mouse models demonstrate that IPH5201 combined with oxaliplatin enhanced the antitumor effect on mice engrafted with murine tumors (5). This effect depended on CD8^+^ and CD4^+^ T-cell activity, indicating that IPH5201 may enhance immunogenic cell death mediated by platinum-based chemotherapy. To follow-up on this hypothesis, IPH5201 is being evaluated in combination with durvalumab and carboplatin or cisplatin with pemetrexed in the phase 2 MATISSE study (NCT05742607) in patients with resectable, stage II-IIIA NSCLC. The expression profile of CD39 in early-stage NSCLC and preclinical combination data further support the clinical evaluation of IPH5201 in combination with durvalumab and chemotherapies in patients with early-stage NSCLC (5).

In summary, findings from this first-in-human, phase 1 study demonstrated that IPH5201 as monotherapy, or in combination with durvalumab, was well tolerated at pharmacologically active doses that induced receptor saturation and reduction of intratumoral CD39 enzymatic activity and provides preliminary evidence for disease stabilization in patients with advanced solid tumors.

## Supplementary Material

Supplementary Appendix Supplementary Appendix and Study Representativeness Figure

Figure S1 Study Design

Figure S2 Treatment with IPH5201 3,000 mg saturates both soluble CD39 in sera and membrane-bound CD39 on immune cells.

Table S1 TEAEs of any grade occurring in >10% of all patients.

Table S2 TRAEs of any grade occurring in >5% of all patients.

Table S3 TEAEs of special interest for IPH5201: thromboembolic events.

Table S4 IPH5201 PK Parameters following a single dose of intravenous IPH5201.

Table S5 Summary of immunogenicity of IPH5201.

## Data Availability

Data underlying the findings described in this article may be obtained in accordance with AstraZeneca’s data sharing policy described at https://www.astrazenecaclinicaltrials.com/our-transparency-commitments/. Data for this study can be requested through Vivli at https://vivli.org/members/enquiries-about-studies-not-listed-on-the-vivli-platform/. The AstraZeneca Vivli member page is also available outlining further details: https://vivli.org/ourmember/astrazeneca/.
