# Supplementary material for: IPH5201, an Anti-CD39 mAb, as Monotherapy or in Combination with Durvalumab in Advanced Solid Tumors
Source: Cancer Res Commun. 2025 Sep 22;5(9):1690–700. doi: 10.1158/2767-9764.CRC-25-0361 (PMC12451260; doi:10.1158/2767-9764.CRC-25-0361)
Supplement: Supplementary Appendix — and Study Representativeness Figure [file crc-25-0361_supplementary_appendix_suppsa.docx]

**IPH5201, an anti-CD39 monoclonal antibody, as monotherapy or in combination with durvalumab in advanced solid tumors**

John Powderly, et al.

**Supplementary Appendix**

**Representativeness of Study Participants**

| Cancer types(s)/stage(s)/ condition | Advanced solid tumors (primarily pancreatic, colorectal, and non-small-cell lung cancers) |
| --- | --- |
| Sex | Cancer impacts men and women equally, though there are sex-specific malignancies. This trial was open to both men and women with advanced solid tumors. Slightly over 50% of patients enrolled in this trial were male (52.6% in the monotherapy group, and 57.9% in the combination therapy group). |
| Race/ethnicity | Cancer impacts all race and ethnic groups, and there are various malignancies that are more common among specific race and/or ethnic groups. This trial was open to patients with advanced solid tumors. In this trial, 97.3% of patients in the monotherapy group and all patients in the combination therapy group were white. |
| Age | Cancer impacts patients of all ages. Some solid tumors are more common with age while others have a predilection for specific decades of life. This trial was open to patients with advanced solid tumors. The median age was 62 years in the monotherapy group and 64 years in the combination therapy group. |
| Geography | Patients were enrolled at 8 study centers in 4 countries (United States, France, Spain, and Switzerland). |
| Overall representativeness of this study | The true distribution of sex, age, race and ethnicity of patients with various advanced solid tumors is unknown. |

**Supplementary Appendix**

**Full eligibility criteria**

***Inclusion criteria***

- Aged ≥18 years at the time of study entry.
- Written informed consent and any locally required authorization (e.g., data privacy) obtained from the patient prior to performing any protocol-related procedures, including screening evaluations.
- Eastern Cooperative Oncology Group Performance Status of 0 or 1.
- Weight ≥35 kg.
- Life expectancy ≥12 weeks, as estimated through The Gustave Roussy Immune Score. Only those patients for whom the Gustave Roussy Immune score is 0 or 1 are deemed eligible for inclusion. The Gustave Roussy Immune score consists of the sum of the following items:
  - Lactate dehydrogenase > upper limit of normal (ULN) = 1.
  - Albumin serum < 35 g/L = 1.
  - Neutrophil-to-lymphocyte ratio > 6 = 1.
- Patients diagnosed with histologically or cytologically confirmed advanced solid tumors.
  - For Part 1 and Part 2 (IPH5201 in monotherapy or combined with durvalumab): Patients must be refractory to standard therapy or for which no standard therapy exists.
  - For pharmacodynamic cohorts: Patients must be diagnosed with advanced squamous cell lung carcinoma or advanced pancreatic ductal adenocarcinoma and have exhausted all the approved standard therapies.
- Adequate organ and marrow function, as defined in the table below.

| **Category** | **Parameter** | **Value** |
| --- | --- | --- |
|  | Hemoglobin ^a^ | ≥9 g/dL |
| Hematological | Absolute neutrophil count ^a^ | ≥1500 µ/L |
|  | Platelet count ^a^ | ≥75,000 µ/L |
|  |  | ≤1.5 × ULN if no demonstrable liver metastases |
|  | Total bilirubin | ≤3 × ULN in the presence of documented Gilbert’s syndrome or liver metastases |
| Hepatic | Alanine transaminase and | ≤2.5 × ULN if no demonstrable liver metastases |
|  | Aspartate transaminase | ≤5 × ULN in the presence of liver metastases |
| Renal | Glomerular filtration rate ^b^ | ≥45 mL/minute |

ULN = upper limit normal.

^a^ Hematological criteria cannot be met with ongoing or recent blood transfusions (within 14 days prior to the scheduled first dose of study treatment) or require growth factor support (within 21 days prior to the scheduled first dose of study treatment).

^b^ As determined by Modification of Diet in Renal Disease Study equation (1).

- Patients must have at least 1 measurable lesion according to Response Evaluation Criteria in Solid Tumors (RECIST) version 1.1.
  - A previously irradiated lesion can be considered a target lesion if the lesion is well defined, measurable per RECIST, and has clearly progressed.
  - Patients without available archival tissue must have a non-target lesion that can be biopsied at acceptable risk (if biopsy is required for enrollment) as judged by the investigator or, if no other lesion is suitable for biopsy, then a RECIST target lesion used for biopsy must be ≥ 2 cm in longest diameter.
- All patients must consent to providing archival tumor specimens for correlative biomarker studies if tumor tissue is available. If an archival specimen is not available, patients must consent to a fresh biopsy.
- For patients enrolled in the pharmacodynamic cohorts, paired fresh tumor biopsies at screening and at Day 15 will be required.
- Females of childbearing potential who are sexually active with a non-sterile male partner must use at least one highly effective method of contraception from the time of screening and must agree to use such precautions for 90 days after the last dose of investigational product. Male partners of a female patient must use a male condom plus spermicide throughout this period. Cessation of birth control contraception after this point should be discussed with a responsible physician. Not engaging in sexual activity for the total duration of the study and the drug washout period is an acceptable practice; however, occasional abstinence, the rhythm method, and the withdrawal method are not acceptable methods of contraception. Female patients should refrain from breastfeeding throughout this period.
- Non-sterile male patients who are sexually active with a female partner of childbearing potential must use a male condom with spermicide from Day 1 through 90 days after receipt of the last dose of investigational product. Periodic abstinence, the rhythm method, and the withdrawal method are not acceptable methods of contraception. It is strongly recommended for the female partner of a male patient to also use a highly effective method of contraception throughout this period. In addition, male patients must refrain from fathering a child or sperm donation while on study and for 90 days after the last dose of investigational product.

***Exclusion criteria***

- Receipt of any conventional or investigational anticancer therapy (anti-CTLA-4, anti-PD-1, anti-PD-L1 antibodies) within 21 days of the planned first dose.
- Receipt of agents targeting CD73, CD39, or adenosine receptors.
- Concurrent enrollment in another therapeutic clinical study. Enrollment in observational studies will be allowed.
- Any toxicity (excluding alopecia) from prior standard therapy that has not been completely resolved to baseline at the time of consent. Patients with Grade 1 or 2 toxicities as defined by the National Cancer Institute Common Terminology Criteria for Adverse Events (NCI CTCAE) version 5.0 that are deemed stable or irreversible can be enrolled on a case-by-case basis with prior consultation and agreement with the medical monitor (e.g., Grade 1 peripheral neuropathy from prior oxaliplatin, Grade 1 skin toxicity from prior cetuximab). For immune-oncology (IO)-pretreated patients:
  - No toxicity leading to permanent discontinuation of prior IO therapy.
  - No grade ≥3 immune-mediated adverse events (imAEs) or an immune-related neurologic or ocular AEs of any grade during prior IO therapy or any unresolved grade ≥1 imAEs.
  - Patients with an endocrine AE of any grade are permitted to enroll if they are stable on appropriate replacement therapy and are asymptomatic.
  - Patients must not have required the use of additional immunosuppression other than corticosteroids for the management of an AE, must not have experienced recurrence of an AE if re-challenged, and must not currently require maintenance doses of >12 mg prednisone or equivalent per day.
- Active or prior documented autoimmune or inflammatory disorders within the past 5 years prior to the start of treatment. The following are exceptions to this criterion:
  - Vitiligo or alopecia.
  - Hypothyroidism (e.g., following Hashimoto syndrome) stable and on hormone replacement.
  - Any chronic skin condition, including psoriasis, that does not require systemic therapy.
  - Celiac disease controlled by diet alone.
- Known allergy or hypersensitivity to any component of investigational product formulations.
- Cardiac and vascular criteria:
  - Mean QT interval corrected for heart rate using Fridericia’s formula (QTcF) ≥ 470 ms calculated from three electrocardiograms (within 5 minutes at 1 minute apart, manually read).
  - Presence of acute coronary syndrome, including myocardial infarction or unstable angina pectoris, other arterial ischemic or thrombotic event, including cerebrovascular accident or transient ischemic attack, within 6 months prior to enrollment.
  - New York Heart Association class II or greater congestive heart failure, serious cardiac arrhythmia requiring medication, or uncontrolled hypertension (>160 mmHg systolic and/or >100 mmHg diastolic), despite appropriate antihypertensive medication.
  - History of hypertensive crisis/hypertensive encephalopathy within the past 6 months prior to the scheduled first dose of study treatment.
  - History of any grade of venous or arterial thromboembolic events within 6 months prior to enrollment.
- Active infection, including:
  - Tuberculosis: clinical evaluation that includes clinical history, physical examination and radiographic findings, and tuberculosis testing in line with local practice.
  - Hepatitis B virus (HBV): known positive HBV surface antigen result.
  - Hepatitis C virus.
  - Human immunodeficiency virus (HIV): documented HIV infection (HIV 1/2 antibody positive). Note the following exceptions:
    - Patients are eligible if CD4 count is over 350 cells/mm^3^ and they are on an anti-retroviral regimen with evidence of at least two undetectable viral loads within the past 6 months on this same regimen; the most recent undetectable viral load must be within the past 12 weeks.
    - For patients who received chemotherapy in the past 6 months, a CD4 count of < 350 cells/mm^3^ during chemotherapy is permitted if viral loads were undetectable during this same chemotherapy.
    - Patients must not be currently receiving prophylactic therapy for an opportunistic infection and must not have had an opportunistic infection within the past 6 months.
- Uncontrolled intercurrent illness, including but not limited to, ongoing or active infection, interstitial lung disease, uncontrolled diabetes, serious chronic gastrointestinal conditions associated with diarrhea, or psychiatric illness/social situations that would limit compliance with study requirements, substantially increase risk of incurring AEs, or compromise the ability of the patient to give written informed consent.
- Other invasive malignancy within 2 years. Noninvasive malignancies (i.e., cervical carcinoma in situ, in situ prostate cancer, non-melanomatous carcinoma of the skin, or ductal carcinoma in situ of the breast that has been surgically cured) are excluded from this definition.
- Untreated central nervous system (CNS) metastatic disease, leptomeningeal disease, or cord compression.
  - NOTE: Patients previously treated for CNS metastases who were radiographically and clinically stable for at least 28 days and who did not require corticosteroids (of any dose) for symptomatic management for at least 14 days prior to the first dose of investigational product are not excluded. Patients whose brain metastases have been treated may participate, provided they showed radiographic stability (radiographic stability is defined as one brain image, obtained after treatment to the brain metastases; this imaging scan should show no evidence of intracranial progression). In addition, any neurologic symptoms that developed either because of the brain metastases or their treatment must have resolved or be stable either without the use of steroids or on a steroid dose of ≤ 12 mg/day of prednisone or its equivalent for at least 14 days prior to the start of treatment.
- Current or prior use of immunosuppressive medication within 14 days prior to the first dose. NOTE: The following are exceptions to this criterion:
  - Intranasal, topical, inhaled corticosteroids, or local steroid injections (e.g., intraarticular injection).
  - Systemic corticosteroids at physiologic doses not to exceed 12 mg/day of prednisone or equivalent.
  - Steroids as premedication for hypersensitivity reactions (e.g., computed tomography [CT] scan premedication).
- Major surgery (as defined by the investigator) within 28 days prior to first dose or still recovering from prior surgery. NOTE: Local procedures (e.g., placement of a systemic port, core needle biopsy, and prostate biopsy) are allowed if completed at least 24 hours prior to the administration of the first dose of study treatment.
- Female patients who are pregnant or breastfeeding, as well as male or female patients of childbearing potential who are not willing to employ one highly effective method of birth control from screening to 90 days after the last dose of investigational product.
- Patients who are incarcerated or are unable to willingly provide consent or are unable to comply with the protocol procedures.
- Any condition that, in the opinion of the investigator or sponsor, would interfere with safe administration or evaluation of the investigational product(s), interpretation of patient safety, or study results.
- Receipt of live, attenuated vaccine within 28 days prior to the first dose of investigational product(s). NOTE: Patients, if enrolled, should not receive live vaccine during the study and 180 days after the last dose of investigational product(s). NOTE: Vaccination with a killed vaccine is permitted at any time.
- Involvement in the planning and/or conduct of the study (applies to both sponsor staff and/or staff at the study site).

**Dose-limiting toxicities (DLTs)**

***AEs considered to be DLTs***

- Any Grade 4 imAE.
- Any Grade ≥3 noninfectious colitis.
- Any Grade ≥3 noninfectious pneumonitis, irrespective of duration.
- Any Grade 3 imAE, other than colitis or pneumonitis, that does not downgrade to Grade 2 within 3 days after onset of the event despite optimal medical management, including systemic corticosteroids, or does not downgrade to Grade ≥1 or baseline within 14 days.
- Any Grade 2 noninfectious pneumonitis that does not resolve to Grade ≥1 within 7 days of the initiation of maximal supportive care.
- Any Grade 3 or 4 hematologic toxicity as described below:
  - Neutropenia:
    - Grade 4 neutropenia, regardless of duration.
    - Grade 3 neutropenia that does not improve to Grade 1 within 7 days of onset, occurring after appropriate granulocyte colony-stimulating factor therapy.
    - Any febrile neutropenia.
  - Anemia:
    - Grade 3 anemia, if associated with clinical sequelae or requires transfusion of >2 units of red blood cells.
    - Grade 4 anemia, regardless of duration.
  - Thrombocytopenia:
    - Grade 3 thrombocytopenia lasting for >7 days.
    - Grade 3 thrombocytopenia, regardless of duration, associated with Grade 3 or higher hemorrhage.
    - Grade 4 thrombocytopenia of any duration.
- Liver Function:
  - For Part 1: Isolated aspartate transaminase (AST) or alanine transaminase (ALT) elevation >5 × ULN if baseline was normal or >10 × ULN if baseline was abnormal or isolated total bilirubin (TBL) >3 × ULN if baseline was normal or >8 × ULN if baseline was abnormal, that does not downgrade to ≤2.5 × ULN if baseline was normal or ≤5 × ULN if baseline was abnormal within 14 days after onset with optimal medical management, including systemic corticosteroids.
  - For Part 2: Isolated AST or ALT elevation >8 × ULN if baseline was normal or >12 × ULN if baseline was abnormal or isolated TBL >5 × ULN if baseline was normal or >10 × ULN if baseline was abnormal, that does not downgrade to ≤2.5 × ULN if baseline was normal or ≤5 × ULN if baseline was abnormal within 14 days after onset with optimal medical management, including systemic corticosteroids.
  - Isolated Grade 4 liver transaminase elevation or TBL, regardless of duration.
  - Any increase in AST or ALT > 3 × ULN and concurrent increase in TBL >2 × ULN (Hy’s Law) without evidence of cholestasis or alternative explanations (e.g., viral hepatitis, disease progression in the liver).
- Any other Grade ≥3 non-imAE, except for the exclusions listed below.

***The DLT definition excludes the following conditions***:

- Grade 3 or 4 lymphopenia of any duration that is not of clinical significance or does not require intervention.
- Grade 3 inflammatory reaction attributed to a local antitumor response (e.g., inflammatory reaction at sites of metastatic disease, lymph nodes, etc.) that resolves to Grade ≤ 1 within 28 days after onset.
- The component of Grade 3 cytokine release syndrome that involves prolonged signs or symptoms lasting ≤ 6 hours from onset, despite maximal supportive care.
- Grade 3 lymph node pain, despite maximal supportive care, lasting ≤ 24 hours.
- Grade 3 diarrhea, nausea, or vomiting that responds to maximal supportive care and improves by at least 1 grade within 3 days after onset.
- Grade 3 fatigue lasting ≤7 days.
- Grade 3 endocrine disorder (thyroid, pituitary, and/or adrenal insufficiency) that is managed with or without systemic corticosteroid therapy and/or hormone replacement therapy, and the patient is asymptomatic.
- Concurrent vitiligo or alopecia of any AE grade.
- Isolated Grade 3 elevations in amylase and/or lipase that are not associated with clinical signs or symptoms or radiographic features suggestive of pancreatitis.
- Isolated Grade 3 electrolyte abnormalities that are not associated with clinical signs or symptoms and are reversed with appropriate maximal medical intervention within 3 days after onset.
- Grade 3 fever lasting ≤ 24 hours with or without medical therapy and is not considered an SAE.

ImAEs are defined as AEs of an immune nature (i.e., inflammatory) in the absence of a clear alternative etiology. In the absence of a clinically significant abnormality, repeat laboratory testing will be conducted to confirm significant laboratory findings prior to designation as a DLT. Based on the emerging safety profile, an AE not listed above may be defined as a DLT after consultation with the sponsor and the Dose-Escalation Committee.

**ELISA quantification of IPH5201 in human serum**

Serum samples for quantification of IPH5201 were collected at treatment cycle 1 on days 1, 2, 8, and 15, then on cycles 2–6 on day 1. All samples were collected pre-dose, except for on cycle 1 day 1 and cycle 3 day 1 when samples were also collected post-dose. Serum samples were pre-diluted at a minimum of 1:10 before incubation on an anti-IPH5201 Fab antibody-coated 96-well plate. IPH5201-bound antibodies were detected using a biotin-conjugated anti-IPH5201 antibody and horseradish peroxidase-conjugated streptavidin. Tetramethylbenzidine substrate was added to the mix, and the peroxidase catalyzed a chemical reaction resulting in a colorimetric change. After stopping the colorimetric reaction with stop solution, the intensity of the color was measured at a wavelength of 450 nm using a spectrophotometer. IPH5201 was used as a reference standard. The study samples were assayed by batch with one calibration curve (15.0 [anchor calibrator], 20.0, 37.5, 75.0, 150, 300, 600, 800 and anchor calibrators 1000 and 1500 ng of IPH5201 per mL) and at least two sets of quality controls (QC) (50.0, 150 and 600 ng of IPH5201 per mL). The concentrations of analyte in calibration standards, QCs and samples were determined using a five-parameter logistic curve-fit calculated from mean OD_nm_ versus concentrations in ng/mL. The zero standard was not included in the calculation. When IPH5201 concentration in the sample was out of range of the calibration curve, the sample was reanalyzed after appropriate dilution according to the validated method. QCs prepared at a concentration higher than the upper limit of quantitation were added to the assay after undergoing the same dilution as the samples.

**Detection of anti-IPH5201 antibodies by electroluminescence**

Serum samples for detection of anti-drug antibodies (ADA) were collected pre-dose on day 1 of treatment cycles 1, 2, 4, 6, and every 12 weeks thereafter. A screening assay was initially used to assess the presence of ADA in clinical samples. Samples which tested positive for the presence of ADA were then tested in a confirmatory assay to demonstrate that ADA were specific for IPH5201. Samples that tested positive in the confirmatory assay were further characterized in a titration assay. The screening, confirmatory and titration assays were performed following the same method. Serum samples were pretreated with acetic acid to dissociate drug-ADA complexes and ADA were extracted on plates coated with the drug and then eluted. After pretreatment, samples were incubated with a mix of biotinylated drug (BIO-IPH5201) and ruthenylated drug (TAG-IPH5201). The mix was then transferred to a blocked streptavidin-coated 96-well plate and incubated to capture the drug-ADA-drug immunocomplexes onto the assay plate via the BIO-IPH5201. Bound complexes were detected using the MSD Meso QuickPlex SQ 120 reader (RRID: SCR_020304) to measure light emitted by TAG-IPH5201 upon electrochemical stimulation. The study samples were assayed by batch with at least 2 sets of QCs that consisted of human serum spiked with anti-IPH5201 antibody at 50.0 and 5000 ng/mL. The signal of each QC and sample was determined as the mean signal of one preparation tested in duplicate.

**CD39 enzymatic assay methods**

Matched pairs of tumor specimens were obtained at screening and during therapy (day 22 ± 1) and immersed in 10% neutral-buffered formalin. After 2 to 7 days, they were transferred to 15% sucrose for up to 12 hours, then to 30% sucrose for 12-72 hours, for cryoprotection. They were then snap-frozen in Optimal Cutting Temperature medium using an isopentane bath cooled by liquid nitrogen, then kept at -80°C until ready to section. Samples were cryosectioned in batches at 5 µm, then fixed in acetone at -20°C for 10 minutes before being immersed in 10% neutral buffered formalin for 2 minutes. Slides were washed in phosphate buffered saline (PBS) 3 times for 2 minutes before being incubated for 15 minutes in 2 mL/slide of a solution containing 50 mM tris maleate, 2 mM calcium chloride and 250 mM sucrose in pure water adjusted to pH 7.4 (solution 1). Slides were then incubated in 2 mL/slide of solution 1 with freshly added 1 mM adenosine triphosphate (ATP), 5 mM manganese chloride, 2 mM lead II nitrate, 2.5% Dextran T200 and 2.5 mM levamisole hydrochloride for 1 hour at 37°C (solution 2). Slides were then washed in PBS before incubation in a freshly prepared solution of 0.5% ammonium sulfide in pure water (solution 3) for 1 minute. The slides were then immersed in 3 changes of water to stop the reaction, counterstained with hematoxylin, dehydrated and permanently cover-slipped. For each sample, a negative control slide was run in addition to the test slide, using solution 1 prepared without ATP. For each staining run, positive controls were included, consisting of previously tested xenograft tumor samples shown to have CD39 expression and enzyme activity. The slides were later scanned to whole-slide images using an Aperio scanner (Leica Biosystems, RRID: SCR_021256) at 40x magnification. For assay evaluation, the biopsies were assessed by a pathologist with experience in enzyme assay development and evaluation. Only patients that had matching pairs of pre- and on-treatment biopsies with tumor tissue were assessed. For each section, enzyme activity (evidenced as brown staining) was evaluated separately in the tumor cell, stromal and vascular components. The pathologist provided a judgement as to whether staining was meaningfully different between pre- and on-treatment samples, based on staining within the different compartments, focusing on the stromal and vascular compartments, known to be the sites of CD39 expression.

**CD39 receptor occupancy**

Serum samples from IPH5201-treated patients were used for the quantification of soluble CD39 via ELISA, and membrane-bound CD39 was quantified on monocytes and B cells isolated from patients via flow cytometry.

**Supplementary Reference**

1. Levey AS, Stevens LA, Schmid CH, Zhang YL, Castro AF 3rd, Feldman HI, *et al*. A new equation to estimate glomerular filtration rate. *Ann Intern Med*. 2009 May 5;**150**(9):604-12. doi: 10.7326/0003-4819-150-9-200905050-00006. Erratum in: *Ann Intern Med*. 2011 Sep 20;155(6):408.
