# Supplementary figures and images for: IPH5201, an Anti-CD39 mAb, as Monotherapy or in Combination with Durvalumab in Advanced Solid Tumors
Source: Cancer Res Commun. 2025 Sep 22;5(9):1690–700. doi: 10.1158/2767-9764.CRC-25-0361 (PMC12451260; doi:10.1158/2767-9764.CRC-25-0361)

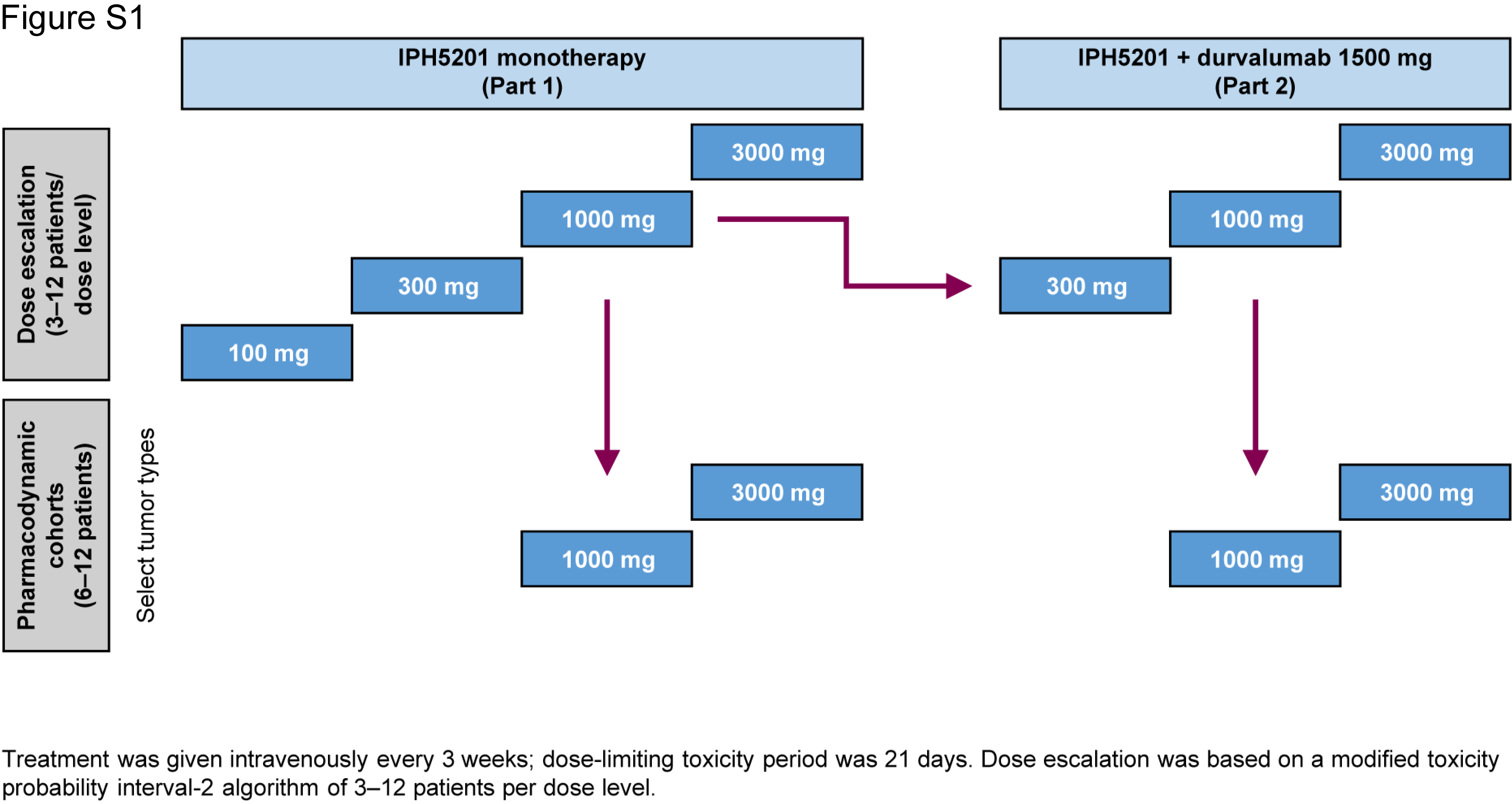

Supplement: Figure S1 — Study Design [file crc-25-0361_figure_s1_suppsf1.png]

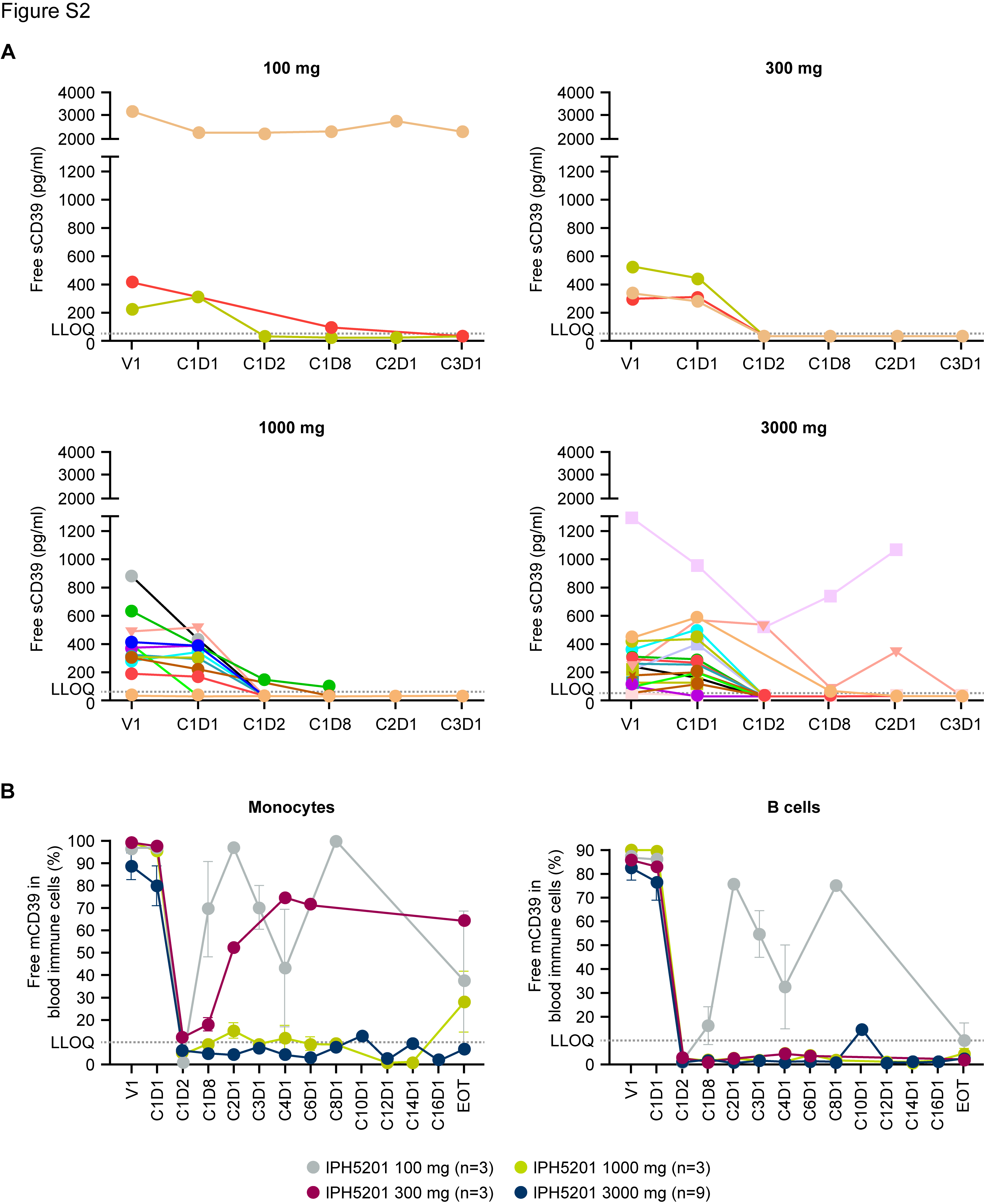

Supplement: Figure S2 — Treatment with IPH5201 3,000 mg saturates both soluble CD39 in sera and membrane-bound CD39 on immune cells. [file crc-25-0361_figure_s2_suppsf2.png]
