# Supplementary material for: IPH5201, an Anti-CD39 mAb, as Monotherapy or in Combination with Durvalumab in Advanced Solid Tumors
Source: Cancer Res Commun. 2025 Sep 22;5(9):1690–700. doi: 10.1158/2767-9764.CRC-25-0361 (PMC12451260; doi:10.1158/2767-9764.CRC-25-0361)
Supplement: Table S1 — TEAEs of any grade occurring in >10% of all patients. [file crc-25-0361_table_s1_suppst1.docx]

**Table S1: TEAEs of any grade occurring in >10% of all patients.^a^**

|  | **IPH5201** | | | | | **IPH5201 + durvalumab 1500 mg** | | | |  |
| --- | --- | --- | --- | --- | --- | --- | --- | --- | --- | --- |
| **TEAEs, n (%)^b^** | **100 mg (n=3)** | **300 mg (n=3)** | **1000 mg (n=13)** | **3000 mg (n=19)** | **Total (N=38)** | **300 mg (n=4)** | **1000 mg (n=8)** | **3000 mg (n=7)** | **Total (N=19)** | **TOTAL**  **(N=57)** |
| **Fatigue** | 0 | 0 | 1 (7.7) | 9 (47.4) | 10 (26.3) | 1 (25.0) | 2 (25.0) | 3 (42.9) | 6 (31.6) | 16 (28.1) |
| **Decreased appetite** | 1 (33.3) | 0 | 2 (15.4) | 4 (21.1) | 7 (18.4) | 2 (50.0) | 2 (25.0) | 4 (57.1) | 8 (42.1) | 15 (26.3) |
| **Infusion-related reaction** | 0 | 1 (33.3) | 4 (30.8) | 3 (15.8) | 8 (21.1) | 0 | 1 (12.5) | 3 (42.9) | 4 (21.1) | 12 (21.1) |
| **Anemia** | 1 (33.3) | 1 (33.3) | 3 (23.1) | 4 (21.1) | 9 (23.7) | 1 (25.0) | 0 | 1 (14.3) | 2 (10.5) | 11 (19.3) |
| **Tumor pain** | 1 (33.3) | 1 (33.3) | 1 (7.7) | 5 (26.3) | 8 (21.1) | 0 | 2 (25.0) | 1 (14.3) | 3 (15.8) | 11 (19.3) |
| **Abdominal pain** | 0 | 0 | 3 (23.1) | 3 (15.8) | 6 (15.8) | 1 (25.0) | 2 (25.0) | 0 | 3 (15.8) | 9 (15.8) |
| **Asthenia** | 0 | 0 | 3 (23.1) | 3 (15.8) | 6 (15.8) | 1 (25.0) | 1 (12.5) | 1 (14.3) | 3 (15.8) | 9 (15.8) |
| **Dyspnea** | 1 (33.3) | 0 | 2 (15.4) | 5 (26.3) | 8 (21.1) | 0 | 1 (12.5) | 0 | 1 (5.3) | 9 (15.8) |
| **Nausea** | 0 | 1 (33.3) | 2 (15.4) | 2 (10.5) | 5 (13.2) | 2 (50.0) | 1 (12.5) | 1 (14.3) | 4 (21.1) | 9 (15.8) |
| **Anxiety** | 0 | 0 | 3 (23.1) | 2 (10.5) | 5 (13.2) | 1 (25.0) | 1 (12.5) | 1 (14.3) | 3 (15.8) | 8 (14.0) |
| **Arthralgia** | 0 | 1 (33.3) | 2 (15.4) | 3 (15.8) | 6 (15.8) | 1 (25.0) | 0 | 1 (14.3) | 2 (10.5) | 8 (14.0) |
| **Constipation** | 1 (33.3) | 0 | 2 (15.4) | 3 (15.8) | 6 (15.8) | 0 | 0 | 2 (28.6) | 2 (10.5) | 8 (14.0) |
| **Cough** | 1 (33.3) | 0 | 2 (15.4) | 2 (10.5) | 5 (13.2) | 1 (25.0) | 0 | 2 (28.6) | 3 (15.8) | 8 (14.0) |
| **Pyrexia** | 0 | 1 (33.3) | 2 (15.4) | 1 (5.3) | 4 (10.5) | 1 (25.0) | 1 (12.5) | 2 (28.6) | 4 (21.1) | 8 (14.0) |
| **Aspartate aminotransferase increased** | 0 | 0 | 3 (23.1) | 1 (5.3) | 4 (10.5) | 2 (50.0) | 1 (12.5) | 0 | 3 (15.8) | 7 (12.3) |
| **Headache** | 0 | 0 | 1 (7.7) | 0 | 1 (2.6) | 1 (25.0) | 2 (25.0) | 2 (28.6) | 5 (26.3) | 6 (10.5) |
| **Pruritus** | 1 (33.3) | 1 (33.3) | 1 (7.7) | 2 (10.5) | 5 (13.2) | 0 | 0 | 1 (14.3) | 1 (5.3) | 6 (10.5) |

^a^Data are shown based on the as-treated population, defined as all subjects who received any investigational product.

^b^Preferred terms were coded as per MedDRA version 25.0.

MedDRA, Medical Dictionary for Regulatory Activities; TEAEs, treatment-emergent adverse events.
