# Supplementary material for: IPH5201, an Anti-CD39 mAb, as Monotherapy or in Combination with Durvalumab in Advanced Solid Tumors
Source: Cancer Res Commun. 2025 Sep 22;5(9):1690–700. doi: 10.1158/2767-9764.CRC-25-0361 (PMC12451260; doi:10.1158/2767-9764.CRC-25-0361)
Supplement: Table S2 — TRAEs of any grade occurring in >5% of all patients. [file crc-25-0361_table_s2_suppst2.docx]

**Table S2: TRAEs of any grade occurring in >5% of all patients.^a^**

|  | **IPH5201** | | | | | **IPH5201 + durvalumab 1500 mg** | | | |  |
| --- | --- | --- | --- | --- | --- | --- | --- | --- | --- | --- |
|  | **100 mg (n=3)** | **300 mg (n=3)** | **1000 mg (n=13)** | **3000 mg (n=19)** | **Total (N=38)** | **300 mg (n=4)** | **1000 mg (n=8)** | **3000 mg (n=7)** | **Total (N=19)** | **TOTAL**  **(N=57)** |
| **Any TRAEs, n (%)**^b,c^ | 1 (33.3) | 3 (100) | 8 (61.5) | 12 (63.2) | 24 (63.2) | 3 (75.0) | 5 (62.5) | 6 (85.7) | 14 (73.7) | 38 (66.7) |
| Infusion-related reaction | 0 | 1 (33.3) | 4 (30.8) | 3 (15.8) | 8 (21.1) | 0 | 1 (12.5) | 3 (42.9) | 4 (21.1) | 12 (21.1) |
| Fatigue | 0 | 0 | 0 | 6 (31.6) | 6 (15.8) | 0 | 2 (25.0) | 2 (28.6) | 4 (21.1) | 10 (17.5) |
| Nausea | 0 | 1 (33.3) | 2 (15.4) | 1 (5.3) | 4 (10.5) | 0 | 1 (12.5) | 0 | 1 (5.3) | 5 (8.8) |
| Arthralgia | 0 | 0 | 1 (7.7) | 3 (15.8) | 4 (10.5) | 1 (25.0) | 0 | 0 | 1 (5.3) | 5 (8.8) |
| Tumor pain | 1 (33.3) | 1 (33.3) | 0 | 1 (5.3) | 3 (7.9) | 0 | 1 (12.5) | 1 (14.3) | 2 (10.5) | 5 (8.8) |
| Pruritus | 1 (33.3) | 1 (33.3) | 1 (7.7) | 2 (10.5) | 5 (13.2) | 0 | 0 | 0 | 0 | 5 (8.8) |
| Asthenia | 0 | 0 | 1 (7.7) | 1 (5.3) | 2 (5.3) | 0 | 1 (12.5) | 1 (14.3) | 2 (10.5) | 4 (7.0) |
| Pyrexia | 0 | 1 (33.3) | 0 | 0 | 1 (2.6) | 0 | 1 (12.5) | 2 (28.6) | 3 (15.8) | 4 (7.0) |
| Decreased appetite | 0 | 0 | 0 | 1 (5.3) | 1 (2.6) | 1 (25.0) | 1 (12.5) | 1 (14.3) | 3 (15.8) | 4 (7.0) |
| Flushing | 0 | 0 | 0 | 2 (10.5) | 2 (5.3) | 0 | 1 (12.5) | 1 (14.3) | 2 (10.5) | 4 (7.0) |

^a^Data are shown based on the as-treated population, defined as all subjects who received any investigational product.

^b^TRAEs may be related to IPH5201, durvalumab or both.

^c^Preferred terms were coded as per MedDRA version 25.0.

MedDRA, Medical Dictionary for Regulatory Activities; TRAEs, treatment-related adverse events.
