# Supplementary material for: IPH5201, an Anti-CD39 mAb, as Monotherapy or in Combination with Durvalumab in Advanced Solid Tumors
Source: Cancer Res Commun. 2025 Sep 22;5(9):1690–700. doi: 10.1158/2767-9764.CRC-25-0361 (PMC12451260; doi:10.1158/2767-9764.CRC-25-0361)
Supplement: Table S3 — TEAEs of special interest for IPH5201: thromboembolic events. [file crc-25-0361_table_s3_suppst3.docx]

**Table S3. TEAEs of special interest for IPH5201: thromboembolic events.^a^**

|  | **IPH5201** | | | | | **IPH5201 + durvalumab 1500 mg** | | | |  |
| --- | --- | --- | --- | --- | --- | --- | --- | --- | --- | --- |
| **TEAEs, n/N (%)^b^** | **100 mg** | **300 mg** | **1000 mg** | **3000 mg** | **Total** | **300 mg** | **1000 mg** | **3000 mg** | **Total** | **TOTAL** |
| **Thromboembolic events**  **Total**  **PDAC**  **Other** | 0/3  0/1  0/2 | 0/3  0/0  0/3 | 3/13 (23.1)  3/9 (33.3)  0/4 | 4/19 (21.1)  3/8 (37.5)  1/11 (9.1) | 7/38 (18.4)  6/18 (33.3)  1/20 (5.0) | 1/4 (25.0)  0/1  1/3 (33.3) | 2/8 (25.0)  2/5 (40.0)  0/3 | 1/7 (14.3)  0/0  1/7 (14.3) | 4/19 (21.1)  2/6 (33.3)  2/13 (15.4) | 11/57 (19.3)  8/24 (33.3)  3/33 (9.1) |
| **Cerebrovascular accident**  Total  PDAC  Other | 0/3  0/1  0/2 | 0/3  0/0  0/3 | 0/13  0/9  0/4 | 0/19  0/8  0/11 | 0/38  0/18  0/20 | 0/4  0/1  0/3 | 1/8 (12.5)  1/5 (20.0)  0/3 | 0/7  0/0  0/7 | 1/19 (5.3)  1/6 (16.7)  0/13 | 1/57 (1.8)  1/24 (4.2)  0/33 |
| **Embolism**  Total  PDAC  Other | 0/3  0/1  0/2 | 0/3  0/0  0/3 | 0/13  0/9  0/4 | 2/19 (10.5)  1/8 (12.5)  1/11 (9.1) | 2/38 (5.3)  1/18 (5.6)  1/20 (5.0) | 1/4 (25.0)  0/1  1/3 (33.3) | 0/8  0/5  0/3 | 1/7 (14.3)  0/0  1/7 (14.3) | 2/19 (10.5)  0/6  2/13 (15.4) | 4/57 (7.0)  0/24  3/33 (9.1) |
| **Portal vein thrombosis**  Total  PDAC  Other | 0/3  0/1  0/2 | 0/3  0/0  0/3 | 0/13  0/9  0/4 | 0/19  0/8  0/11 | 0/38  0/18  0/20 | 0/4  0/1  0/3 | 1/8 (12.5)  1/5 (20.0)  0/3 | 0/7  0/0  0/7 | 1/19 (5.3)  1/6 (16.7)  0/13 | 1/57 (1.8)  1/24 (4.2)  0/33 |
| **Pulmonary embolism**  Total  PDAC  Other | 0/3  0/1  0/2 | 0/3  0/0  0/3 | 3/13 (23.1)  3/9 (33.3)  0/4 | 2/19 (10.5)  2/8 (25.0)  0/11 | 5/38 (13.2)  5/18 (27.8)  0/20 | 0/4  0/1  0/3 | 0/8  0/5  0/3 | 0/7  0/0  0/7 | 0/19  0/6  0/13 | 5/57 (8.8)  5/24 (20.8)  0/33 |

^a^Data are shown based on the as-treated population, defined as all subjects who received any investigational product.

^b^Preferred terms were coded as per MedDRA version 25.0.

MedDRA, Medical Dictionary for Regulatory Activities; PDAC, pancreatic ductal adenocarcinoma; TEAEs, treatment-emergent adverse events.
