# Supplementary material for: IPH5201, an Anti-CD39 mAb, as Monotherapy or in Combination with Durvalumab in Advanced Solid Tumors
Source: Cancer Res Commun. 2025 Sep 22;5(9):1690–700. doi: 10.1158/2767-9764.CRC-25-0361 (PMC12451260; doi:10.1158/2767-9764.CRC-25-0361)
Supplement: Table S4 — IPH5201 PK Parameters following a single dose of intravenous IPH5201. [file crc-25-0361_table_s4_suppst4.docx]

**Table S4: IPH5201 PK Parameters following a single dose of intravenous IPH5201.^a^**

|  | **IPH5201** | | | | **IPH5201 + durvalumab 1500 mg** | | |
| --- | --- | --- | --- | --- | --- | --- | --- |
| **PK Parameter** | **100 mg (n=3)** | **300 mg (n=3)** | **1000 mg (n=13)** | **3000 mg**  **(n=19)** | **300 mg**  **(n=4)** | **1000 mg**  **(n=8)** | **3000 mg**  **(n=7)** |
| **Single dose** | | | | | | | |
| Cmax, μg/mL | 18.88 (10.41) | 64.37 (13.31) | 339.1 (39.56) | 1121 (27.37) | 88.92 (28.58) | 524.7 (47.71) | 1227 (45.14) |
| t_max_, day (min–max) | 0.08 (0.0632–0.0910) | 0.05 (0.0521–0.139) | 0.07 (0.0583–0.158) | 0.06 (0.0556–0.146) | 0.06 (0.0576–0.0694) | 0.07 (0.0542–1.08) | 0.06 (0.0549–0.119) |
| AUC_(0–21)_, day* μg/mL | NC | 213.3 (42.20) | 2162 (34.93) | 7514 (20.95) | 345.7 (13.47) | 3094 (31.95) | 8381 (41.06) |
| t_1/2ʎz_, day | NC | 2.609 (44.59) | 8.434 (27.96) | 9.269 (24.69) | 3.856 (39.48) | 8.697 (62.67) | 9.176 (12.79) |
| C_trough_, μg/mL | NC | NC | 33.43 (58.91) | 140.6 (37.02) | 0.9239 (151.6) | 43.98 (68.89) | 172.6 (57.25) |
| CL, L/day | NC | 1.392 (44.13) | 0.3815 (39.61) | 0.3159 (24.55) | 0.8729 (11.40) | 0.2498 (50.88) | 0.3108 (39.21) |
| V_Z_, L | NC | 5.238 (12.87) | 4.642 (32.31) | 4.225 (27.40) | 4.856 (40.22) | 3.135 (22.71) | 4.115 (34.97) |

Geometric mean (CV%) presented, except for t_max_, where median (min–max) is shown.

^a^Data are shown based on the PK analysis set, defined as patients who received ≥1 dose of IPH5201 or durvalumab and provided ≥1 quantifiable post-treatment sample.

AUC_inf_, area under the serum concentration-time curve from zero to infinity; AUC_last_, area under the serum concentration-time curve from zero to the last quantifiable concentration; AUC_last_/D, dose-normalized AUC_last_; CL, total body clearance of drug from serum after intravascular administration; C_max_, maximum observed (peak) drug concentration; C_trough_, observed lowest drug concentration reached before the next dose is administered; CV%, percent coefficient of variation; NA, not applicable; NC, not calculated; PK, pharmacokinetic; R_ac_, accumulation ratio for C_max_/C_trough_, t_max_, time to reach peak or maximum observed concentration following drug administration; t_1/2ʎz_, half-life associated with terminal slope of a semi-logarithmic concentration-time curve; V_Z_, volume of distribution following iv administration (based on terminal phase).
