# Supplementary material for: IPH5201, an Anti-CD39 mAb, as Monotherapy or in Combination with Durvalumab in Advanced Solid Tumors
Source: Cancer Res Commun. 2025 Sep 22;5(9):1690–700. doi: 10.1158/2767-9764.CRC-25-0361 (PMC12451260; doi:10.1158/2767-9764.CRC-25-0361)
Supplement: Table S5 — Summary of immunogenicity of IPH5201. [file crc-25-0361_table_s5_suppst5.docx]

**Table S5: Summary of immunogenicity of IPH5201.^a^**

|  | **IPH5201** | | | | | **IPH5201 + durvalumab 1500 mg** | | | |  |
| --- | --- | --- | --- | --- | --- | --- | --- | --- | --- | --- |
| **ADA Category, n (%)** | **100 mg (n=3)** | **300 mg (n=3)** | **1000 mg (n=13)** | **3000 mg**  **(n=18)** | **Total**  **(N=37)** | **300 mg**  **(n=4)** | **1000 mg**  **(n=8)** | **3000 mg**  **(n=5)** | **Total**  **(N=17)** | **TOTAL**  **(N=54)** |
| ADA positive at any time (ADA prevalence)^b^ | 3 (100) | 3 (100) | 8 (61.5) | 11 (61.1) | 25 (67.6) | 4 (100) | 7 (87.5) | 1 (20.0) | 12 (70.6) | 37 (68.5) |
| Treatment-emergent ADA positive (ADA incidence)^c^ | 3 (100) | 3 (100) | 8 (61.5) | 7 (38.9) | 21 (56.8) | 4 (100) | 7 (87.5) | 1 (20.0) | 12 (70.6) | 33 (61.1) |
| Treatment-boosted ADA^d^ | 0 | 0 | 0 | 0 | 0 | 0 | 0 | 0 | 0 | 0 |
| Treatment-induced ADA (positive post-baseline only) | 3 (100) | 3 (100) | 8 (61.5) | 7 (38.9) | 21 (56.8) | 4 (100) | 7 (87.5) | 1 (20.0) | 12 (70.6) | 33 (61.1) |
| ADA positive at baseline only | 0 | 0 | 0 | 1 (5.6) | 1 (2.7) | 0 | 0 | 0 | 0 | 1 (1.9) |
| ADA positive post-baseine and positive at baseline | 0 | 0 | 0 | 3 (16.7) | 3 (8.1) | 0 | 0 | 0 | 0 | 3 (5.6) |
| Persistently positive^e^ | 2 (66.7) | 1 (33.3) | 1 (7.7) | 2 (11.1) | 6 (16.2) | 2 (50.0) | 1 (12.5) | 0 | 3 (17.6) | 9 (16.7) |
| Transiently positive^f^ | 1 (33.3) | 2 (66.7) | 7 (53.8) | 5 (27.8) | 15 (40.5) | 2 (50.0) | 6 (75.0) | 1 (20.0) | 9 (52.9) | 24 (44.4) |

^a^Data are shown based on the ADA-evaluable population, defined as patients who received ≥1 dose of IPH5201 or durvalumab and provided baseline and ≥1 post-treatment sample.

^b^ADA prevalence: percentage of participants with positive ADA result at any time, baseline or post-baseline.

^c^Treatment-emergent ADA: either treatment-induced ADA or treatment-boosted ADA; ADA incidence is the percentage of participants who were treatment-emergent ADA positive.

^d^Treatment-boosted ADA: a positive ADA titer at baseline that was boosted to ≥4 fold during the study period.

^e^Persistently positive: being treatment-emergent ADA positive and having ≥2 post-baseline ADA positive measurements with >16 weeks between the first and last positive.

^f^Transient positive: being treatment-emergent ADA positive and having ≥1 post-baseline ADA positive measurement and not fulfilling the conditions for persistently positive.
